# Homeostatic microbiome disruption as a cause of insulin secretion disorders. *Candida albicans*, a new factor in pathogenesis of diabetes: A STROBE compliant cross-sectional study

**DOI:** 10.1097/MD.0000000000031291

**Published:** 2022-11-11

**Authors:** Dragan M. Nikolic, Vesna Dimitrijevic-Sreckovic, Lazar T. Ranin, Milos M. Stojanovic, Iva D. Ilic, Drasko M. Gostiljac, Ivan A. Soldatovic

**Affiliations:** a Faculty of Medicine Belgrade, University of Belgrade, Belgrade, Serbia; b Clinic for Endocrinology, Diabetes and Metabolic Diseases, University Clinical Centre of Serbia, Belgrade, Serbia; c Institute of Microbiology and Immunology, Belgrade, Serbia; d Institute of Public Health of Serbia “Dr. Milan Jovanovic Batut”, Belgrade, Serbia; e Institute of Medical Statistics and Informatics, Belgrade, Serbia.

**Keywords:** *Candida albicans*, *Diphtheroids*, *Enterobacter sp*, homeostatic microbiome, insulin secretion, pathogenesis of diabetes, *Pseudomonas aeruginosa*, *Staphylococcus aureus*

## Abstract

The study aimed to test the hypothesis that homeostatic microbiome (HM) disorders lead to the increased indirect influence of certain microorganisms (MO) in the gastrointestinal tract, causing a disorder of insulin secretion, insulin resistance, and diabetes. We highlighted *Candida* and certain types of bacteria since previous in vitro research showed they significantly affect insulin secretion and can cause insulin resistance in obese patients with metabolic syndrome. After determining the type of MO present in the throat swab and the stool, the oral glucose tolerance test (OGTT) test, and analysis of glucose and insulin secretion were performed in patients (n = 38) who were positive for certain types of MO compared to negative patients. Finally, all patients were divided into two groups: overweight patients (body mass index [BMI] < 30) and obese patients (BMI > 30). These two groups were compared for the percentage of certain types of MO to determine which MO can affect an increase in obesity and BMI. The presence of *Diphtheroids* in the throat (60.5%) reduces insulin secretion in patients compared with the negative group (194.5: 332.4) and the difference was statistically significant (*P* = .030). The presence of *Candida* in the throat (10%) increases insulin secretion, but the difference was statistically insignificant. The presence of *Candida* in the stool (28.9%) also increases insulin secretion and the difference was statistically significant (*P* = .038). Cumulative results (throat + stool) were similar (180: 332, *P* = .022). Analysis of BMI showed that the percentage of *Diphtheroids* in the throat decreases with increased body weight (53.8: 75%) while the percentage of *Candida* (38.5: 8.3%) and *Enterobacter* (61.5: 25%) increases, but these differences were statistically insignificant (*P* > .05). *Diphtheroids* in the throat can reduce insulin secretion by synthesizing their metabolites. *Candida albicans* is a conditional pathogen and as a significant indirect factor induces increased insulin secretion and insulin resistance. There are indications that elevated levels of *Candida* in the intestinal system can cause increased body weight of patients. *C albicans* should be considered a new factor in the pathogenesis of diabetes.

## 1. Introduction

Type 2 diabetes is a persistent and in the initial phase almost imperceptible disease that causes serious clinical manifestations over the years. It is a result of two interrelated problems – insulin resistance and insulin secretion disorder.^[1]^ Several factors are involved in the etiopathogenesis of this disease: physical inactivity, overweight,^[2]^ genetic factors, malnutrition in the fetal and prenatal period, and some drugs (e.g., diuretics, anti-hypertensives, steroids). The risk of type 2 diabetes increases with increased adipose tissue storage in obese people. Adipose tissue produces adiponectin, tumor necrosis factor (TNF), alpha, leptin, resistin, and IL-6, thereby affecting insulin resistance and possibly pancreatic β-cell dysfunction.^[3–5]^ New factors in the etiopathogenesis of diabetes mellitus include microbiological agents, due to direct infection of the pancreas, or saprophytic flora disorder.^[8]^ Homeostatic microbiome (HM) is a set of all microorganisms (bacteria, fungi, and viruses) which by their mutual relationship and action maintain the normal homeostasis of the organism.^[6]^

Our in vitro studies conducted in pancreatic islet culture,^[7,8]^ have shown that certain types of bacteria and fungi can directly cause increased or decreased insulin secretion of the pancreas leading to insulin resistance and the development of diabetes mellitus. Based on these results, the following hypothesis was presented – the presence of these microorganisms in the human pancreas can lead to insulin resistance, thus increasing the chances of development of type 2 diabetes mellitus, or they can cause β-cell destruction due to activation of the immune system in response to infection, thus inducing type 1 diabetes mellitus.^[6]^

Patients with metabolic syndrome were followed in this study. Metabolic syndrome includes central (abdominal) obesity, some disorders of glucoregulation diabetes mellitus type 2, impaired fasting glucose (IFG) and impaired glucose tolerance (IGT), hypertension, and hyperlipoproteinemia.^[9]^ Metabolic syndrome was determined according to the recommendations of the National Cholesterol Education Program (NCEP).^[10]^ In particular, the most important risk factors for metabolic syndrome are abdominal obesity and insulin resistance.^[11]^ In obese individuals, proinflammatory cytokines and hormones are released from adipose tissue and participate in the development of insulin resistance.^[12]^ Insulin resistance is characterized by reduced sensitivity of peripheral tissues to insulin, which leads to the development of hyperinsulinemia.^[13]^ Lipotoxicity is also present in obese people, due to the accumulation of lipid precursors which are toxic to cells.^[14]^

**Figure 1.**
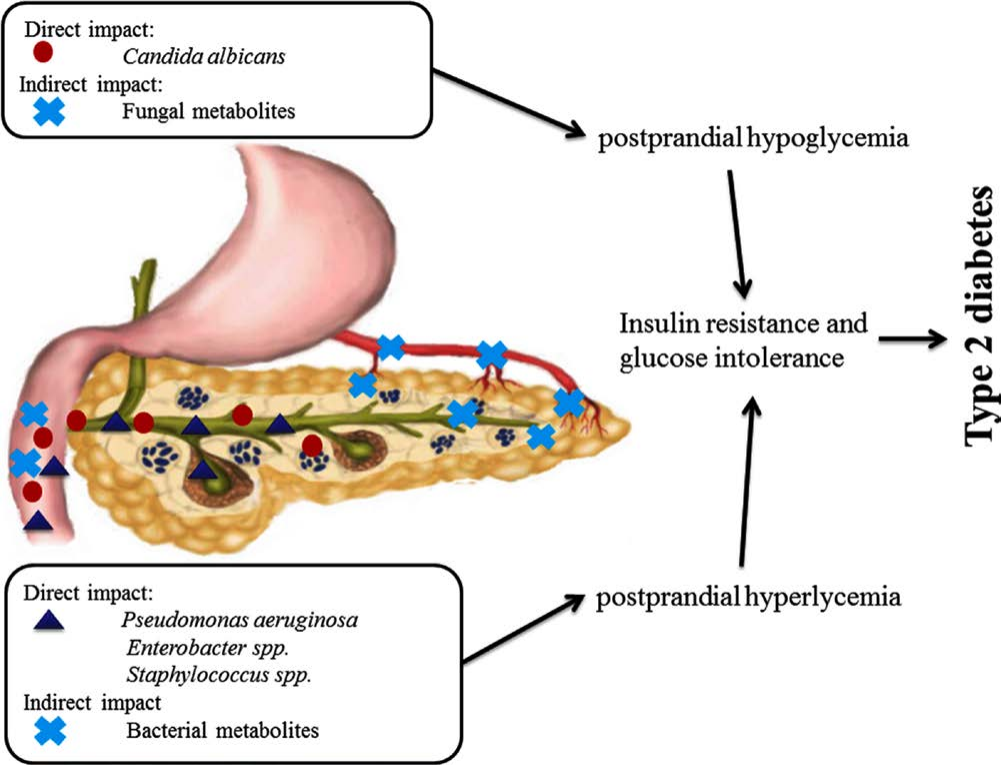
The direct and indirect influence of *Candida* and bacteria on the development of diabetes mellitus type 2. (Nikolic DM. Military-medical and pharmaceutical review 2018;75(11):1110–1117).

My previous in vitro studies have shown that certain strains of microorganisms can provoke increased or decreased insulin secretion. *Candida* in pancreatic islet culture induces increased insulin secretion.^[8]^ while certain strains of bacteria, such as *Pseudomonas*, *Staphylococcus*, and *Enterobacter* reduce insulin secretion.^[7]^ Based on these results, the hypothesis was presented that microorganisms by their action can cause insulin resistance, a prerequisite for the development of diabetes (Fig. 1).

The second part of the hypothesis refers to the indirect action of microorganisms: Due to HM disorder, overgrowth of these microorganisms in the intestinal system and mucous surfaces can indirectly affect insulin secretion. They release certain metabolites that through blood can reach the pancreas.^[6]^ This hypothesis was tested in a pilot program with patients who have metabolic syndrome and insulin resistance. The aim was to determine the strains of microorganisms that cause these effects in vivo, in patients.

## 2. Material and Methods

Study design: The research was designed to determine first the type of microorganisms (MO) present in stool and throat swabs of all patients. After that, the oral glucose tolerance test (OGTT) test and the analysis of glucose and insulin secretion were performed in patients who were positive for certain types of MO compared to negative patients. Finally, all patients were divided into two groups. The first group is overweight patients (body mass index [BMI] < 30) and the second group is obese patients (BMI > 30). These two groups were compared for the percentage of certain types of MO to determine MO that can affect the increase in obesity and BMI.

**Figure 2.**
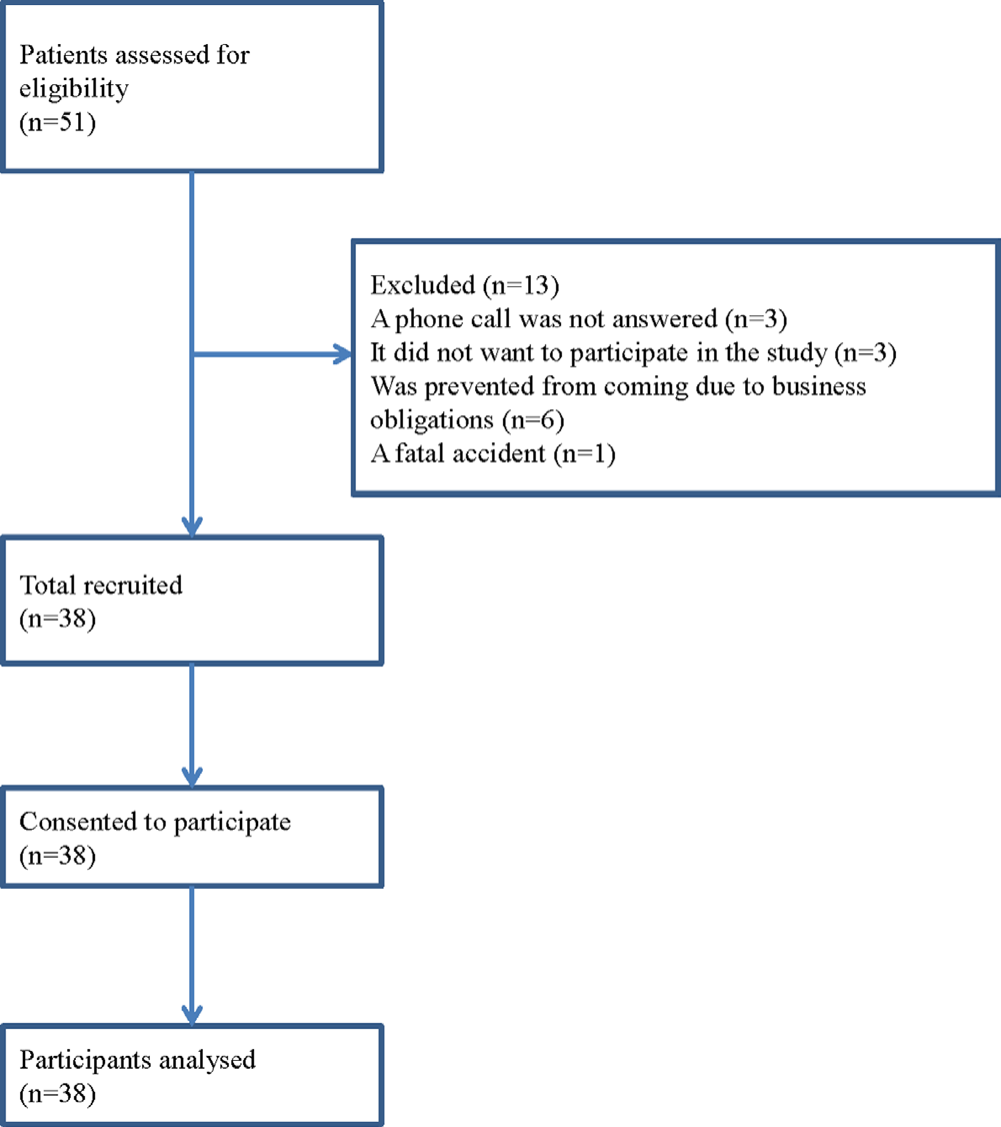
Flow diagram of study participation.

Throat swabs and stool specimens were taken from patients (36 women and 2 men, mean age 60.6 ± 10.4, min = 34, max = 72) who came to the Cabinet for Nutrition and Prevention of Metabolic Disorders at the Clinic for Endocrinology, Diabetes, and Metabolic Diseases, University Clinical Center of Serbia in Belgrade, due to problems with overweight, in the period from 2017 to 2020 (Fig. 2).

Participants were informed about the purpose of this research and gave their written consent for voluntary participation in this study, so it is conducted following the decision of the Ethics Committee of the Medical Faculty in Belgrade (No. 29/XII-10). This study follows the principles of the Declaration of Helsinki.

All patients who participated in this study were obese Caucasians from the territory of Belgrade and had different professions. All parameters to determine the BMI of patients were obtained. Anthropometric measurements: body weight, body height, body mass index, waist circumference, hip circumference, and waist to hip circumference ratio, were performed. Body mass index was calculated as body weight in kilograms divided by the square of the body height in meters. Glucose regulation was tested by OGTT which was performed on fasting patients by drinking 75 gr of glucose dissolved in 300 mL of water. Then the values of glycemia and insulin were measured at 0, 30, and 120 minutes. Insulin levels were determined by radioimmunoassay microbiological analyses of samples (stool and throat swabs) were performed by standard procedure at the Institute for Microbiology, Clinical Center in Serbia. Microbiological isolates were identified based on microscopic, cultural, and biochemical properties. Microscopic slides were prepared directly from patient samples, and also from colonies on culture plates, and then Gram stained. We used Columbia blood agar plates (7% sheep blood), MacConkey agar and XLD agar plates, and Sabourand dextrose agar. Depending on the cultural and morphological characteristics of isolated microorganisms, we have prepared a small series of biochemical tests.

In this cross-sectional study, results are presented as count (%), means ± standard deviation, or median (25^th^–75^th^ Percentile) depending on data type and distribution. Groups are compared using parametric (t-test) and nonparametric (Pearson Chi-square test, Fisher exact test, Mann–Whitney U test) tests. All *P* values less than .05 were considered significant. All data were analyzed using SPSS 20.0 (IBM Corp. Released 2011. IBM SPSS Statistics for Windows, Version 20.0. Armonk, NY: IBM Corp.).

## 3. Results

Microbiological analysis of throat swab samples detected seven different types of microorganisms in different percentages (Table 1). Most abundant is *Staphylococcus aureus* (97.4%), followed by *Streptococcus alfa haemolyticus* (92%), *Diphtheroids* (60%), and *Neisseria sp* (44.7%). The least represented are *Moraxela cathoralis* and *Candida albicans* with 15.8% and 10.5% respectively.

**Table 1:** Types of microorganisms detected in the throat and stool swabs of the patients. Example: Out of a total number of examined patients, *Candida* was detected in stool samples of 11 subjects, which makes up about 28.9%.

	N (%)	Mean percentage representation of positive samples
Throat		
*Diphtheroids*	23 (60.5%)	12.6
*Staphylococcus aureus*	37 (97.4%)	28.7
*Moraxella catarrhalis*	6 (15.8%)	30.0
*Streptococcus pneumonie*	9 (23.7%)	31.1
*Streptococcus viridans*	35 (92.1%)	37.7
*Neisseria* sp.	17 (44.7%)	22.9
*Candida albicans*	4 (10.5%)	17.5
Stool		
*Escherichia coli*	33 (86.8%)	53.2
*Enterococcus* sp.	16 (42.1%)	16.2
*Citrobacter* sp.	9 (23.7%)	29.4
*Candida albicans*	11 (28.9%)	31.8
*Morganella morganii*	6 (15.8%)	23.3
*Proteus* sp.	10 (26.3%)	30.5
*Pseudomonas aeruginosa*	6 (15.8%)	15.0
*Klepsiella* sp.	4 (10.5%)	20.0
*Alcaligenes faecalis*	1 (2.6%)	20.0
*Enterobacter* sp.	19 (50%)	29.7
*Providencia stuartii*	1 (2.6%)	30.0
*Diphtheroids*	1 (2.6%)	20.0
*Streptococcus* sp.	2 (5.3%)	15.0
*Serratia* sp.	2 (5.3%)	15.0
*Bacillus* sp.	1 (2.6%)	10.0
*Providencia rettgeri*	1 (2.6%)	30.0
Throat + stool		
*Candida albicans*	13 (34.2%)	32.3

N (%) = count (percent).

Microbiological analysis of stool specimens detected 16 microorganisms. The most abundant are *Escherichia coli* and *Enterococcus* sp. 86.8% and 42.1%, respectively. *Klepsiela*, *Pseudomonas aeryginosa*, *Morganela morganini*, *Citrobacter*, *Proteus sp*, *C albicans* are present in 10% to 30%. Other microorganisms are present in less than 10% (Table 1). Mean values of detected microorganisms are shown in the right column of Table 1.

The dependence of glucose AUC (area under curve) on the type of MO present in the sample is shown in Table 2. The results show that certain MO does not influence glucose AUC. The values obtained for a positive and negative group of patients are very similar and there is no statistical significance between them. This applies to both types of samples, throat swabs and stool specimens (Table 2).

**Table 2:** Glucose AUC in patients positive or negative for a specific type of MO (No and Yes groups). No statistically significant differences were detected (*P* > .05).

	NO		YES		*P* value
	N	Mean ± SD	N	Mean ± SD	
Throat					
*Diphtheroids*	15	30.9 ± 5.6	23	29.6 ± 5.8	.751
*Staphylococcus aureus*	1	27.7	37	30.2 ± 5.8	
*Moraxella catarrhalis*	32	30.1 ± 6.1	6	30.7 ± 2.5	.958
*Streptococcus pneumonie*	29	30.4 ± 5.3	9	29.5 ± 7.2	.539
*Streptococcus viridans*	3	24.7 ± 6.4	35	30.6 ± 5.5	
*Neisseria*sp.	21	30.8 ± 5.4	17	29.5 ± 6.1	.596
*Candida albicans*	34	29.8 ± 5.5	4	33.4 ± 7.3	.391
Stool					
*Escherichia coli*	5	32.8 ± 6.1	33	29.8 ± 5.6	.641
*Enterococcus* sp.	22	30.2 ± 6.1	16	30.1 ± 5.2	.971
*Citrobacter* sp.	29	29.7 ± 6.2	9	31.6 ± 3.3	.641
*Candida albicans*	27	31.1 ± 4.8	11	27.8 ± 7.2	.114
*Morganella morganii*	32	30.1 ± 5.7	6	30.9 ± 6.0	.744
*Proteus* sp.	28	29.6 ± 6.2	10	31.8 ± 4.0	.354
*Pseudomonas aeruginosa*	32	30.4 ± 5.2	6	29.1 ± 8.5	.541
*Klepsiella* sp.	34	30.4 ± 5.7	4	28.1 ± 6.4	.569
*Alcaligenes faecalis*	37	30.1 ± 5.8	1	34.4	
*Enterobacter* sp.	19	30.3 ± 5.5	19	30.1 ± 6.1	.846
*Providencia stuartii*	37	30.3 ± 5.7	1	24.7	
*Diphtheroids*	37	29.9 ± 5.6	1	38.7	
*Streptococcus* sp.	37	30.1 ± 5.8	1	33.2	
*Serratia* sp.	36	30.2 ± 5.8	2	29.8 ± 3.8	
*Bacillus* sp.	36	30.5 ± 5.6	2	23.7 ± 5.8	
*Providencia rettgeri*	37	30.3 ± 5.7	1	24.6	
Throat + stool					
*Candida albicans*	25	31.4 ± 4.8	13	27.8 ± 6.6	.069

AUC = area under curve, Mean = arithmetic mean, MO = microorganisms, N = count, SD = standard deviation.

Results for the influence of MO on insulin AUC are given in Table 3. A comparison of positive and negative groups of patients (presence or absence of certain types of MO, respectively) showed that patients with *Diphtheroids* in the throat had a lower total insulin secretion (194.5: 332.4) and the difference was statistically significant (*P* ≥ .030). Patients with *Candida* in the stool had increased insulin secretion compared to negative patients (374.1: 199) and an increase was statistically significant (*P* = .038).

**Table 3:** Insulin AUC in patients positive or negative for a specific type of MO (No and Yes groups). Statistically significant differences were detected in patients positive for *Diphtheroids* in the throat, *Candida albicans*, and *Proteus* sp in the stool, and in patients positive for *Candida albicans* in both (throat + stool) samples (*P* ≤ .05).

	NO		YES		*P* value
	N	Med (25–75th perc.)	N	Med (25–75th perc.)	
Throat					
*Diphtheroids*	15	332.4 (179.8–514.3)	23	194.5 (142.8–361.7)	.030
*Staphylococcus aureus*	1	374.1	37	199.8 (153.9–390.4)	–
*Moraxella catarrhalis*	32	214.475 (164.05–384.8)	6	182.575 (145.9–390.4)	.598
*Streptococcus pneumonie*	29	199.4 (145.9–366.5)	9	305.5 (157.7–395.5)	.379
*Streptococcus viridans*	3	194.5 (153.95–305.5)	35	211.2 (145.9–395.5)	.644
*Neisseria* sp.	21	211.2 (153.95–366.5)	17	199.4 (170.4–437.4)	.908
*Candida albicans*	34	199.4 (145.9–390.4)	4	327.2 (260.7–494.8)	.216
Stool					
*Escherichia coli*	5	374.1 (153.95–657.1)	33	199.85 (157.7–366.5)	.331
*Enterococcus* sp.	22	267.3 (170.4–390.4)	16	196.95 (128–363.975)	.271
*Citrobacter* sp.	29	211.2 (170.4–395.5)	9	177.9 (142.9–374.1)	.543
*Candida albicans*	27	199.0 (142.9–361.7)	11	374.1 (194.5–555.4)	.038
*Morganella morganii*	32	205.525 (155.825–382.25)	6	246.175 (142.9–437.4)	.953
*Proteus* sp.	28	290 (174.15–404.7)	10	166.9 (122.55–211.2)	.037
*Pseudomonas aeruginosa*	32	242.85 (155.825–392.95)	6	180.075 (142.8–229.1)	.493
*Klepsiella* sp.	34	220.15 (170.4–395.5)	4	149.925 (129.95–257.825)	.167
*Alcaligenes faecalis*	37	211.2 (157.7–390.4)	1	142.9 (142.9–142.9)	–
*Enterobacter* sp.	19	199.85 (142.9–332.45)	19	321.95 (153.95472.2)	.311
*Providencia stuartii*	37	211.2 (157.7–390.4)	1	142.8 (142.8–142.8)	–
*Diphtheroids*	37	199.85 (153.95–374.1)	1	437.4 (437.4–437.4)	–
*Streptococcus* sp.	37	199.85 (153.95–374.1)	1	413.9 (413.9–413.9)	–
*Serratia* sp.	36	220.15 (155.825–392.95)	2	172.875 (145.9–199.85)	.518
*Bacillus* sp.	36	205.525 (155.825–392.95)	2	244.05 (114–374.1)	.640
*Providencia rettgeri*	37	199.85 (153.95–374.1)	1	472.2 (472.2–472.2)	–
Throat + stool					
*Candida albicans*	25	180.3 (142.90–361.70)	13	332.45 (199.40–514.35)	.022

AUC = area under curve, Med = median, MO = microorganisms, N = count, Perc = percentile.

Analysis of cumulative results (both throat + stool samples) showed that patients with *Candida* in both samples had an increased AUC compared to the negative group (332: 180.3) and the difference was statistically significant *P* = .022. For other detected MO, the differences between the two groups were not statistically significant (*P* ≥ .05).

This study included the influence of MO on BMI (Table 4). Patients were divided into two groups. The first group consists of 12 subjects with a BMI < 30 (overweight patients) and the second group of 26 subjects with a BMI > 30 (obese patients). A comparison of both groups regarding the type of MO present in the throat or stool, showed no statistically significant differences, *P* > .05.

**Table 4:** Comparison of the percentage of certain microorganisms in overweight patients (BMI < 30) and obese patients (BMI > 30). No statistical difference present (*P* > .05).

	BMI		*P* value
	<30 [N (%)]	30 + [N (%)]	
Throat			
*Diphtheroids*	9 (75.0%)	14 (53.8%)	.294
*Staphylococcus aureus*	12 (100%)	25 (96.2%)	1.000
*Moraxella catarrhalis*	0	6 (23.1%)	.179
*Streptococcus pneumonie*	2 (16.7%)	7 (26.9%)	.689
*Streptococcus viridans*	12 (100%)	23 (88.5%)	.538
*Neisseria*sp.	5 (41.7%)	12 (46.2%)	1.000
*Candida albicans*	2 (16.7%)	2 (7.7%)	.577
Stool			
*Escherichia coli*	11 (91.7%)	22 (84.6%)	1.000
*Enterococcus*sp.	5 (41.7%)	11 (42.3%)	1.000
*Citrobacter*sp.	2 (16.7%)	7 (26.9%)	.689
*Candida albicans*	1 (8.3%)	10 (38.5%)	.121
*Morganella morganii*	2 (16.7%)	4 (15.4%)	1.000
*Proteus*sp.	4 (33.3%)	6 (23.1%)	.694
*Pseudomonas aeruginosa*	3 (25.0%)	3 (11.5%)	.357
*Klepsiella*sp.	1 (8.3%)	3 (11.5%)	1.000
*Alcaligenes faecalis*	1 (8.3%)	0	.316
*Enterobacter*sp.	3 (25.0%)	16 (61.5%)	.079
*Providencia stuartii*	0	1 (3.8%)	1.000
*Diphtheroids*	0	1 (3.8%)	1.000
*Streptococcus*sp.	0	1 (3.8%)	1.000
*Serratia*sp.	1 (8.3%)	1 (3.8%)	.538
*Bacillus*sp.	0	2 (7.7%)	1.000
*Providencia rettgeri*	0	1 (3.8%)	1.000
Throat + stool			
*Candida albicans*	2 (16.7%)	11 (42.3%)	.158

BMI = body mass index.

However, *Candida* was present in a higher percentage in the stool samples of obese than in overweight patients, 38.5% and 8.3% respectively. Similarly, *Enterobacter* was more present in the second (61.5%) than in the first group (25%). No statistically significant difference was likely detected due to the small number of participants (*P* = .121, *P* = .079).

## 4. Discussion

Previous in vitro studies in pancreatic islet culture have shown that *Pseudomonas aeruginosa, Enterobacter sp*, and *S aureus* decrease insulin secretion while *C albicans* increases insulin secretion, so this section will pay particular attention to these microorganisms and their effects in the studied patients. A new microorganism, *Diphtheroids* has also appeared in the throat that has not been detected in the pancreas and pancreatic islets culture.

*Diphtheroids* found in the patient’s throat probably represent a milder form of infection causing a mild inflammatory reaction. After *S aureus* (97.4%) and *Streptococcus alpha hemolyticus* (92%), they are the third most abundant bacteria by 60% (Table 1). Subjects positive for *Diphtheroids* showed no signs of severe infections and were not treated with any antibiotic therapy. *Diphtheroids* belong to the phylum actinobacteria. They live in commensal relationships with both humans and animals. Corynebacterium is part of the human saliva microbiome.^[15]^ Corynebacterium species is widespread, it is found in soil, water, plants, and what is important for us in food products. Non-diphtheroid species can occur in the mucous membranes and skin flora of humans and animals.^[16,17]^ In clinical practice, attention is generally paid to infectious strains. Some species of *Corynebacterium* are used for industrial production, for instance, *Corynebacterium glutamicum* is used for the production of amino acids, glutamic acid, and lysine, which are used in food and pharmaceutical products. The most significant pathogen of *Coryneform* bacteria is *Corynebacterium diphtheriae*, the primary cause of diphtheria. It is an acute and contagious infection characterized by pseudomembranes of dead epithelial cells, white blood cells, red blood cells, and fibrin formed around the tonsils and the back of the throat.^[18]^ The literature often states that this phenomenon is caused by debilitated host immunity, which is not true. Shifting from commensal to a pathogen form can be a consequence of the disrupted relationship between the microorganisms and the increased number of some MOs at the expense of others. Or it is a dislocation of certain strains to places where they express their pathogenicity. In this study, only the genus (*Corynobacterium*), but no particular species were determined. Nonpathogenic species of *Corynebacteria* such as *Corynebacteria glutamycym* produce glutamic acid, which is, as monosodium-glutamate, used in the production of yogurt and soy sauce.^[19]^ How complex the relationship between bacteria and their struggle for dominance in the mucosa is shown by the fact that some species produce metabolites similar to antibiotics: bacteriocins such as corynecin-linocins antitumor agents.^[20,21]^ Glutamate as a derivative of glutamic acid has significant participation in the body’s metabolism and is also known as a neurotransmitter. In animal studies, the addition of glutamic acid dimethyl ester mainly enhances insulin release at an intermediate glucose concentration in the rat pancreas.^[22]^ L-glutamine alone failed to stimulate proinsulin biosynthesis or insulin release in rat pancreatic islets.^[23]^ Subjects positive for *Diphtheroids* in the throat had a statistically significant, lower total insulin secretion, 194.5: 332.4 (Table 3). However, in the literature, there is no evidence that diphtheroid’s metabolites may affect the reduction of insulin secretion in human research models. No diphtheroid infections have been reported in patients with diabetes.^[24]^ It was also observed that with increasing body weight, the percentage of diphtheroid in the throat decreases (75:53%) (Table 4). The glucose AUC analysis showed no differences between Diphtheroid positive and negative groups (Table 2).

In our study, *Candida* was detected in the throat and stool of 10% and 30% of the patients, respectively (Table 1). In Table 3 there are two statistically significant results: patients who had stool candidiasis had increased insulin secretion compared to negative patients (374.1: 199). Regarding cumulative results (stool + throat samples), patients positive for *Candida* in both samples had increased insulin AUC compared to the negative group (332: 180.3) and the difference was statistically significant *P* = .022. For other detected MOs, the differences were not statistically significant. In our previously published studies, it was established that *C albicans* increases insulin secretion up to seven times in human pancreatic islet culture.^[8]^ In the literature, there are data on the possible influence of *Candida* on the host’s glucose homeostasis in systemic infections. Studies on mice have shown that *Candida* uses pharmacological or genetic agents to affect glucose metabolism and disrupt the host’s glucose homeostasis*. Candida* depletes glucose leading to the rapid death of macrophages.^[25]^ In this particular manuscript, there is an interesting fact. Metformin was administered to healthy mice, but they didn’t have low blood glucose even after long-term administration. However, in the case of infection with small doses of *C albicans*, the same animals had severe hypoglycemia even on the first day of infection. Our research has confirmed that *Candida* increases insulin secretion provoking decreasing in blood glucose levels. Low glucose level enables *Candida* the transition from yeast to hyphal cells, one of the key factors in the virulence and spreading of the disease.^[26]^ However, increased insulin secretion leads to hypoglycemia, which causes hunger and forces the body to take food and sugar. This maintains a nutrient medium that allows the reproduction of *Candida* and its superiority over other MOs in competition for the same ecological niche, intestinal mucosa. The genus *Candida* includes more than 350 species present in humans and other mammals, birds, fish, insects, arthropods, animal waste, plants, and substrates naturally rich in sugars (e.g. honey, nectar, grapes, fermentation, and dairy products), fresh and sea water and airborne particles.^[27]^ *C albicans* is a commensal and a constituent of the normal microflora in 80% of the human population, and predominately colonizes the mucosal surface of the gastrointestinal tract, genitourinary tract, and, to a lesser extent, the skin.^[28,29]^ However, especially in immunocompromised patients (e.g., cancer chemotherapy, AIDS, organ transplantation, or neonates) or when the competing flora are eliminated (e.g., after antibiotic treatment), *C albicans* becomes an opportunistic pathogen that can cause superficial as well as systemic and potentially life-threatening infections.^[30,31]^ During the mucosal invasion, cells of *C albicans* induce glycolytic pathways, tricarboxylic acid cycle, and beta oxidative fatty acid gene. *C albicans* is highly adapted to different environmental conditions thanks to the SAP gene family, with 10 members encoding aspartic proteinases in response to the available nitrogen and carbon source.^[32–34]^ The proposed mechanism of infection includes digestion of host proteins for nutrient supply and degradation of antibodies and complement components/^[35]^ *Candida* has developed several mechanisms that enable successful colonization and evasion of host immune response. This leads to the question of whether the presence of *Candida* in the food is desirable or not. Consumption of sugar-rich food means intake of *Candida* too. Does the presence of *Candida* help degrade sugar, thus contributing to metabolism or it is considered just a pathogenic agent? Why it is advisable to eat raw fruits and tubers during the diet? By consuming organically grown food, microbiomes are also introduced which help the breakdown of that food in the gastrointestinal system. A similar process occurs with the natural rot of fruits and vegetables in nature.^[36]^ In our study, the influence of MO on BMI was examined. Patients were divided into two groups. The first group consisted of patients with BMI < 30 (12 overweight patients), and the second group was patients with BMI > 30 (26 obese patients). A comparison of both groups regarding the presence of certain MO in the throat or the stool, showed no statistically significant differences, *P* > .05. Analysis of results showed that obese patients had a higher percentage of *Candida* in stool samples compared to overweight patients, 38.5% and 8.3% respectively. This indicates that the percentage of *Candida* in an intestinal system increases with increased BMI.

*S aureus* was present in 97% of throat swab samples*. S aureus* is a Gram-positive, anaerobe bacterium, member of *Firmicutes.* This bacteria is usually a commensal of human microbiota often present in the upper respiratory tract and on the skin. It can also become an extracellular opportunistic pathogen, causing localized infections like soft tissue abscesses, but also life-threatening systemic diseases such as infective endocarditis, and meningitis. Pathogenic strains produce exotoxins that damage tissue and protect bacteria from the host’s immune response.^[37,38]^ Previous studies showed that *Staphylococcus* reduces insulin secretion in cell culture. However, in the body, the relationship between bacteria and hosts is much more complex. It is interesting that *S aureus*, exhibits polymorphism in the glucocorticoid receptor gene, resulting in increased corticosteroid production.^[39]^ Corticosteroids have a major effect on the metabolism of the human body. They promote lipolysis and protein catabolism. Glucocorticoids cause a decrease in peripheral glucose uptake and utilization and at the same time stimulate the process of gluconeogenesis in the liver, that is the process of glucose synthesis from non-carbohydrate components, glycerol, and amino acids formed during lipolysis and protein catabolism. Due to this effect of glucocorticoids on carbohydrate metabolism, there is an increase in blood glucose levels. Hyperglycemia leads to increased insulin secretion. Table 3 showed that patients positive for *Staphylococcus* in stool samples (199: 374) had reduced insulin secretion compared to negative patients. These results match the results of the glucose stimulation test, the counterpart for OGTT, in pancreatic islet cell culture,^[7]^ where these bacteria also caused reduced insulin secretion. Cholesterol-lowering therapy may reduce the pathogenicity of *Staphylococcus*, due to similarities in the pathways for staphyloxanthin and human cholesterol biosynthesis.^[40]^ These results are obtained from studies on mice and we don’t know how this will reflect in humans. This type of therapy was not monitored in the examined patients and taken into account.

*Enterobacter sp* was detected in 50% of stool samples (Table 1). A comparison of patients positive or negative for *Enterobacter* (Table 3), showed that bacteria increases insulin secretion (321: 199), while it decreases in cell culture.^[7]^ There are two reasons why. First, there was a small number of examined patients. Second, there are other bacteria present in the microflora that, by their synergistic action, can also change insulin secretion, while in cell culture is present only one type of infection. If *Enterobacter* penetrates and infects the pancreas, it causes a decreased insulin secretion. However, if it overgrows in the intestinal system, it causes increased insulin secretion. In both cases, *Enterobacter* contributes to the development of insulin resistance. *Enterobacter* is a genus of gram-negative, facultatively anaerobic, rod-shaped bacteria that are widely distributed in the environment and is a part of the normal flora of the gastrointestinal tract in 40% to 80% of people. Like most members of the *Enterobacteriaceae*, these organisms are capable of causing opportunistic infection in hospitalized or weakened patients.^[41]^ The urinary and respiratory tract are frequent targets of infection. The genus *Enterobacte*r is a member of the coliform group of bacteria. A recent study suggests that bacteria can contribute to the development of obesity in humans via endotoxin and inflammation. The authors demonstrated that bacteria isolated from morbidly obese patients induce obesity and insulin resistance in germ-free mice.^[42]^ When the abundance of bacteria decreases from 35% in the patient’s gut to non-detectable, weight loss occurs. Strain *Enterobacter cloacae* B29, isolated from the patients, induced obesity and insulin resistance in C57BL/6J-free mice fed a high-fat diet. These data are consistent with our results since with weight gain the percentage of bacteria in the intestinal system increases (Table 4). *Enterobacter* was more prevalent in obese patients than in the overweight group, 61.5% and 25% respectively. Due to the small number of analyzed patients, the difference was not statistically significant (*P* = .079).

*P aeruginosa* was found in 15.8% of stool samples*. P aeruginosa* is a species of great medical importance, it is widespread and is found in soil, water, and on the surface of the skin in humans as well as in residential areas. It can cause sepsis in people with reduced immunity. Lung, kidney, and urinary tract infections can be potentially life-threatening.^[43]^ Due to the lack of oxygen from the substrate, *P aeruginosa* uses nitrates and nitrites, and if there are none, it is capable of fermenting arginine and pyruvate by phosphorylation.^[44]^ *P aeruginosa* creates membrane vesicles (MV) that are released into the culture medium during normal growth. Their release increases approximately threefold after exposure to four times the minimum inhibitory concentration (MIC) of gentamicin. In addition to LPS, several other enzymes (e.g. phospholipase C, protease, hemolysin, and alkaline phosphatase) contribute to the pathogenicity of pseudomonas infections. These vesicles could play an important role in genetic transformation by serving as a transport vehicle for DNA and virulence factors and are likely to be involved in septic shock.^[45]^ Type of diet influence the development of certain microorganisms and their transition from commensal to pathogen form (instant disruption of the homeostatic microbiome). Low levels of phosphate in the human intestinal system activate the transition from benign symbiont to a pathogen and deadly toxins are released in the intestine that can be fatal for the host. This can be alleviated by providing excess phosphate instead of antibiotics.^[46]^ Patients positive for *Pseudomonas* had a lower insulin secretion than the negative group, 180 and 242, 8 respectively (Table 3). These results confirm previously published data,^[7]^ *Pseudomonas* in pancreatic islet cell culture reduces insulin secretion. BMI analysis shows when body weight increases, the percentage of *Pseudomonas* in the intestinal tract of patients decreases (25: 11.5%).

## 5. Conclusion

We examined the direct influence of microorganisms on insulin secretion in cell culture (in vitro) which is parallel to the direct infection of the pancreas in vivo and we concluded the following:

There is an increased number of microorganisms on intestinal mucosa due to the disrupted HM. They secrete their metabolites into the blood, thus affecting the host’s overall metabolism and causing insulin secretion disorder, insulin resistance, and obesity, prerequisites for the development of diabetes.

*Diphtheroids* were present in 60% of throat swab samples. Our study showed that patients having *Diphtheroids* in the throat had lower total insulin secretion. It was also found that with increasing body weight, the percentage of *diphtheroid* in the throat decreases.

The analysis of throat swabs revealed a high percentage of *S aureus* as much as 97%. *Staphylococcus* reduces insulin secretion, but its percentage in the throat does not change with the change in the patient’s body weight.

*Enterobacter* was detected in 50% of stool samples. A comparison of groups of patients positive and negative for *Enterobacter* revealed that bacteria cause increased insulin secretion, while in cell culture they cause decreased insulin secretion.^[7]^ The main reasons are a small number of examined patients and other bacteria present in the microflora that, by their synergistic action, can also change insulin secretion, while in cell culture was only one type of infection. If *Enterobacte*r penetrates and infects the pancreas it will cause a decrease in insulin secretion. However, if it overgrows in the intestinal system it causes an increase in insulin secretion. In both cases, *Enterobacter* contributes to the development of insulin resistance.

*Pseudomonas aeruginosis* was detected in 15.8% of stool samples. Patients positive for *Pseudomonas* had lower insulin secretion compared to the negative group. These results confirm previously published data where *Pseudomonas* reduces insulin secretion in cell culture.^[7]^ Analysis of BMI data revealed that an increase in body weight reduces the percentage of Pseudomonas in the intestinal tract.

The hypothesis that *C albicans* can disrupt insulin secretion, thus leading to diabetes,^[6,8]^ has been confirmed. Namely, in the group of examined subjects, *Candida* was present in 10% of the throat samples and 30% of stool samples. If *Candida* overgrows in the human intestinal system, these patients will undoubtedly have increased insulin secretion during OGTT. This condition undoubtedly leads to insulin resistance. Cumulative results (throat + stool samples) confirm the synergistic effect of *Candida*. Multivariate statistical analyzes of the influence of Candida on insulin secretion (insulin AUC), glucose status (glucose AUC), and the percentage of *Candida* concerning the patient’s body weight showed that:

Patients with *Candida* in the stool had increased insulin secretion (insulin AUC) compared to negative patients (374.1: 199) and an increase was statistically significant (*P* = .038).

Cumulative results (throat + stool samples) showed that patients with *Candida* in both samples had higher insulin AUC compared to the negative group (332: 180.3) and the difference was statistically significant *P* = .022.

Obese patients had a higher percentage of *Candida* in stool samples compared to overweight patients, 38.5% and 8.3% respectively. This indicates that the percentage of *Candida* present in the intestinal system significantly increases with increasing BMI.

It was determined that *C albicans* has direct and indirect effects on insulin secretion and should be seriously taken into account as one of the possible factors involved in the pathogenesis of diabetes.

## Author contributions

DN, II, VDS, and LR collected clinical data. IS, MS, and DG performed the statistical analysis. All authors interpreted the data. DN drafted the first version of the manuscript. VDS, II, LR, IS, MS, and DG critically reviewed the manuscript. All authors have read and approved the final version of the manuscript.

**Conceptualization:** Dragan M. Nikolic.

**Data curation:** Dragan M. Nikolic, Vesna Dimitrijevic-Sreckovic, Lazar T. Ranin, Iva D. Ilic, Ivan A. Soldatovic.

**Formal analysis:** Lazar T. Ranin, Iva D. Ilic.

**Investigation:** Dragan M. Nikolic, Drasko M. Gostiljac.

**Methodology:** Dragan M. Nikolic.

**Software:** Milos M. Stojanovic, Ivan A. Soldatovic.

**Validation:** Dragan M. Nikolic, Vesna Dimitrijevic-Sreckovic, Milos M. Stojanovic, Iva D. Ilic, Drasko M. Gostiljac, Ivan A. Soldatovic.

**Writing – original draft:** Dragan M. Nikolic.
